# Funktionelle Ergebnisse und Wiederaufnahme von Sport, Arbeit und Alltagsaktivitäten nach Revision eines Monoschlittens im Vergleich zur Primärimplantation eines Mono- und Doppelschlittens

**DOI:** 10.1007/s00132-024-04472-z

**Published:** 2024-02-13

**Authors:** Christian B. Scheele, Matthias F. Pietschmann, Thomas C. Wagner, Peter E. Müller

**Affiliations:** 1 Klink für Orthopädie und Unfallchirurgie, Muskuloskelettalen Universitätszentrum München (MUM), Ludwig-Maximilians-Universität, Campus Großhadern, Marchioninistr. 15, 81377 München, Deutschland; 2 Klinik und Poliklinik für Orthopädie und Sportorthopädie, Klinikum rechts an der Isar der Technischen Universität München, Ismaninger Str. 22, 81675 München, Deutschland

**Keywords:** Endoprothetik, Arthrose, Prothesendesign, Revisionschirurgie, Behandlungsergebnis, Arthroplasty, Osteoarthritis, Prothesis design, Revision surgery, Treatment outcome

## Abstract

**Hintergrund:**

Neben dem etablierten Doppelschlitten (bikondylärer Kniegelenksersatz [TKA]) hat sich, bei geeigneter Patientenselektion, der Monoschlitten (unikondylärer Kniegelenksersatz [UKA]) in der operativen Therapie der Gonarthrose bewährt. In klinischen Studien zeigt er überlegene funktionelle Ergebnisse bei geringeren Komplikationsraten. Im klinischen Alltag sind diese Vorteile, insbesondere bei jüngeren, sportlich und beruflich aktiven Patienten gegen den Nachteil einer erhöhten Revisionsrate abzuwiegen. Das Ergebnis einer ggf. früheren Revision erscheint hier relevant.

**Fragestellung:**

Ziel dieser Studie war es, sowohl funktionelles Ergebnis als auch den Zeitraum bis zur Wiederaufnahme von Alltags-, beruflichen und sportlichen Aktivitäten nach Revision eines Mono- auf einen Doppelschlitten denen von primären Mono- und Doppelschlitten anhand einer Matched-Pair-Vergleichsanalyse gegenüberzustellen.

**Methodik:**

Die Studie basierte auf einer Matched-Pair-Vergleichsanalyse zu zwei definierten Zeitpunkten und verglich stets 28 Patienten, die entweder die Revision eines Mono- auf einen Doppelschlitten, eine primäre Implantation eines Monoschlittens oder die eines Doppelschlittens erhielten. Die Patienten beantworteten im Rahmen eines standardisierten Follow-ups den Oxford Knee Score, den UCLA-Score, den Knee Society Score sowie den WOMAC-Score. Darüber hinaus wurden die postoperative Patientenzufriedenheit sowie die Wiederaufnahme von Alltags-, beruflichen und sportlichen Aktivitäten standardisiert erfasst und eine klinische Untersuchung durchgeführt.

**Ergebnisse:**

Die vier untersuchten Funktions-Scores zeigten einen gemeinsamen Trend zugunsten der Monoschlitten, gefolgt von den primären Doppelschlitten und Revisionsdoppelschlitten. Die Unterschiede der Revisionsdoppelschlitten und der primären Doppelschlitten waren hierbei nicht signifikant. Allerdings lagen die Ergebnisse der konvertierten Monoschlitten 3,2 Jahre nach der letzten Operation signifikant unter denen der primären Monoschlitten. Die Rückkehr zur beruflichen und sportlichen Aktivität gelang nach Monoschlitten tendenziell am frühesten, gefolgt von Doppelschlitten und Revisionsgruppe. In allen Gruppen zeigte sich ein Trend zur Durchführung sog. Low-Impact-Sportarten.

**Diskussion:**

Die funktionellen Ergebnisse eines konvertierten Monoschlittens zeigen sich denen der Primärimplantation auf Basis des 3‑Jahres-Follow-ups signifikant unterlegen. Die Rückkehr in Beruf, Sport und Alltag dauerte nach Revision tendenziell länger als nach Primärimplantation eines Mono- oder Doppelschlittens.

## Abstract (en)

**Background:**

Unicompartmental knee arthroplasty (UKA), in addition to total knee arthroplasty (TKA), has been shown to be effective in the surgical treatment of knee osteoarthritis with appropriate patient selection. In clinical studies, it has demonstrated superior functional results with lower complication rates. In clinical practice, these advantages must be weighed against the disadvantage of an increased revision rate, especially in younger patients with sports and work activities.

**Objectives:**

The aim of this study was to compare the functional outcome as well as the time to return to daily activities, work, and sports after revision of UKA to TKA with those of primary UKA and primary TKA using a matched-pair analysis.

**Materials and methods:**

The study was based on a matched-pair analysis at two defined time points, always comparing 28 patients who underwent either revision of a UKA to a TKA, primary UKA, or primary TKA. Patients completed the Oxford Knee Score, UCLA score, Knee Society score, and WOMAC score during standardized follow-up. In addition, postoperative patient satisfaction and return to activities of daily living, work, and sports were recorded in a standardized manner, and a clinical examination was performed.

**Results:**

The four functional scores studied showed a common trend in favor of UKA, followed by primary TKA and revision TKA. The differences between converted UKA and primary TKA were not significant. However, at 3.2 years after the last surgery, the results of the converted UKA were significantly lower than those of the primary UKA. Return to work and sports tended to occur the earliest after UKA, followed by TKA and the revision group. All groups showed a tendency to engage in low-impact sports.

**Conclusion:**

The functional results of revised UKA were significantly inferior to those of primary UKA based on a 3-year follow-up. Return to work, sports, and activities of daily living tended to take longer after revision than after primary implantation of either a UKA or a TKA.

Die Gonarthrose ist weit verbreitet und häufig mit erheblichen Einschränkungen der Alltagsmobilität und Lebensqualität assoziiert. Viele Patienten, die die Indikationskriterien für einen Monoschlitten erfüllen, werden dennoch primär mit einem Doppelschlitten versorgt. Grund sind höhere Versagensraten, die sekundär zum Wechsel auf einen Doppelschlitten führen. Zur Unterstützung der klinischen Entscheidungsfindung vergleicht diese Matched-Pair-Analyse die funktionellen Ergebnisse von je 28 Patienten, die primär mit einem Mono- oder Doppelschlitten versorgt wurden, mit jenen, bei denen die genannte Wechseloperation bereits erfolgte.

## Hintergrund und Fragestellung:

Der Monoschlitten (UKA) hat sich in der Behandlung der anteromedialen Gonarthrose unter Beachtung entsprechender Kontraindikationen etabliert [13, 19, 24]. Als vorteilhaft gegenüber dem klassischen Doppelschlitten (TKA) werden die kürzere Rehabilitationszeit, die geringeren Komplikationsraten sowie überlegene funktionelle Ergebnisse angeführt [18, 23]. Nachteilig zeigen sich höhere Lockerungsraten, wobei im Fall einer Revisionsoperation der Wechsel auf einen Doppelschlitten empfohlen wird [20].

Besonders jüngere, sportlich und beruflich aktive Patienten profitieren von einer beschleunigten beruflichen und privaten Rehabilitation und überlegenen klinischen Ergebnissen. Anderseits erfährt auch ein gesteigertes Risiko einer Wechseloperation angesichts der verbleibenden Lebenserwartung und Anspruchshaltung jüngerer Patienten erhebliche Bedeutung. Häufig steht man im klinischen Alltag vor der Herausforderung, die genannten klinischen Vorteile mit den Nachteilen einer möglichen Wechseloperation abzuwägen.

Die vorliegende Untersuchung der Zufriedenheit, „patient reported outcome measures“ (PROM) sowie Sportfähigkeit und Wiederaufnahme der Alltags- und Berufsaktivität nach Revision eines Monoschlittens im Vergleich zur Primärimplantation eines Mono- und Doppelschlittens soll die Entscheidungsfindung bei dieser Abwägung im klinischen Alltag unterstützen.

## Studiendesign und Untersuchungsmethoden

Das Studiendesign wurde vorab von der Ethikkommission der medizinischen Fakultät genehmigt und sämtliche Patienten gaben nach umfassender Aufklärung und vor Einschluss in die Studie eine schriftliche Einverständniserklärung ab.

Die betrachteten Operationen wurden zwischen 1998 und 2011 durchgeführt. Hierbei wurden folgende drei Gruppen unterschieden, erfasst und ausgewertet:

1. Monoschlitten-Gruppe: Primärimplantation eines unikondylären Kniegelenksersatzes
2. Doppelschlitten-Gruppe: Primärimplantation eines bikondylären Kniegelenksersatzes
3. Revisions-Gruppe: Revision von einem unikondylären Kniegelenksersatz auf einen bikondylären Kniegelenksersatz

Im Zentrum der Untersuchung stand die Revision eines unikondylären auf einen bikondylären Kniegelenksersatz in Gruppe 3. Die Gruppen 1 und 2 wurde auf Basis der Fallzahl (*n* = 28) und der Follow-up-Periode der Revisions-Gruppe, unter Berücksichtigung von Body-Mass-Index (BMI), Geschlecht und Alter im Rahmen einer Matched-Pair-Vergleichsanalyse zusammengestellt. Hierbei wurde beim Matching 1 der Zeitraum zwischen Revisionsoperation und Follow-up der Wechsel-Gruppe (3,2 Jahre) und beim Matching 2 der Zeitraum zwischen Primärimplantation und Follow-up in der Wechsel-Gruppe (6,4 Jahre) zugrunde gelegt, also die Gesamtzeit des „Lebens mit einer Prothese“ im Sinne der Summe der Zeiträume aus Mono- und Doppelschlitten.

Alle Gruppen umfassen 28 Patienten. Merkmale der Patienten wie Alter, Geschlecht, operierte Seite, Größe, Gewicht, BMI und Follow-up-Zeitraum sind Tab. 1 zu entnehmen.

**Table 1:** 

	U2T	Matching 1			Matching 2		
		UKA	TKA	P‑Wert	UKA	TKA	P‑Wert
**Männlich**	11 (39,3 %)	12 (42,9 %)	13 (46,4 %)	*–*	12 (42,9 %)	11 (39,3 %)	*–*
**Weiblich**	17 (60,7 %)	16 (57,1 %)	15 (53,6 %)	*–*	16 (57,1 %)	17 (60,7 %)	*–*
**Seite (rechts/links)**	15/13	11/17	20/8	*–*	15/13	20/8	*–*
**Körpergröße (cm)**	167 SD 7,5 (155–181)	169 SD 10,1 (156–193)	170 SD 8,1 (156–190)	*–*	168 SD 8,9 (155–186)	170 SD 8,0 (156–190)	*–*
**Gewicht (kg)**	82 SD 15,3 (52–115)	82 SD 17,6 (53–140)	84 SD 21,8 (56–144)	*–*	78 SD 11,1 (53–105)	81 SD 19,3 (56–144)	*–*
**Body-Mass-Index**	29,2 SD 4,7 (21,6–39,8)	28,6 SD 6,2 (21,8–56,1)	29,0 SD 6,2 (21,3–45,3)	*0,918* *UKA-TKA: ns TKA-U2T: ns UKA-U2T: ns*	27,5 SD 3,6 (21,1–37,2)	28,1 SD 5,9 (20,2–45,3)	*0,370* *UKA-TKA: ns* *TKA-U2T: ns* *UKA-U2T: ns*
**Alter** **(Primärimplantation)**	61,7 ± 8,5 (42,8–75,3)	–	–	*–*	62,9 ± 8,1 (48,8-80,8)	63,9 ± 9,2 (38,8–82,0)	*0,045* *UKA-TKA: ns TKA-U2T: ns UKA-U2T: ns*
**Alter** **(letzte Operation)**	64,9 SD 9,0 (46,0–77,2)	66,2 SD 5,9 (54,9–79,5)	65,8 SD 7,2 (48,5–77,6)	*0,045* *UKA-TKA: ns* *TKA-U2T: ns* *UKA-U2T: ns*	–	–	*–*
**Follow-up (seit Primärimplantation)**	6,4 ± 3,5 (2,2–16,7)	–	–	*–*	6,2 ± 2,5 (1,5–9,5)	6,0 ± 1,5 (4,3–8,6)	*0,702* *UKA-TKA: ns TKA-U2T: ns UKA-U2T: ns*
**Follow-up** **(seit letzter Operation)**	3,2 SD 1,9 (1,0–8,7)	3,3 SD 1,9 (1,0–8,6)	3,6 SD 1,6 (2,3–8,2)	*0,702* *UKA-TKA: ns* *TKA-U2T: ns* *UKA-U2T: ns*	–	–	*–*
**Alter** **(Follow-up)**	68,1 SD 9,5 (48,8–85,9)	69,5 SD 6,2 (58,6–81,4)	69,4 SD 7,0 (52,9–80,2)	*0,757* *UKA-TKA: ns* *TKA-U2T: ns* *UKA-U2T: ns*	69,1 ± 8,8 (53,5–88,7)	69,9 ± 9,7 (43,1–89,4)	*0,279* *UKA-TKA: ns TKA-U2T: ns UKA-U2T: ns*

*SD* Standardabweichung, *TKA* bikondylärer Kniegelenksersatz, *U2T* Konversion von einem unikondylären Kniegelenksersatz auf einen bikondylären Kniegelenksersatz, *UKA* unikondylärer Kniegelenksersatz

Hinsichtlich der Implantate wurde in Gruppe 1, also im Falle des primären Monoschlittens, das Modell Oxford Phase III (Zimmer Biomet, Warsaw, IN, USA) eingebracht. In den Gruppen 2 und 3, also in allen Fällen eines Doppelschlittens, wurde das Modell Innex Fix CR Fixed Bearing mit einer Inlayhöhe von 10 mm (Zimmer Biomet) verwendet. Alle Implantate wurden in zementierter Technik verankert. Patienten mit anderen Prothesenmodellen, Voroperationen am ipsilateralen Kniegelenk oder endoprothetischem Gelenkersatz am kontralateralen Kniegelenk wurden von der Studie ausgeschlossen. Die operative Versorgung und die postoperative Nachbehandlung erfolgten bei allen Patienten vergleichbar.

Zu Beginn der Studie wurde allen Patienten ein Katalog mit Fragen bezüglich Zufriedenheit, allgemeinem Gesundheitszustand, Berufstätigkeit, Alltagsaktivität und Sportfähigkeit sowie vier etablierte orthopädische Funktions-Scores (PROM) zugesandt. Letztere umfassten den Oxford Knee Score [5], den UCLA-Score [29], den Knee Society Score (KSS) [10] und den WOMAC-Score [3, 26].

Der Oxford Knee Score besteht aus 12 Multiple-Choice-Fragen mit je 5 Antwortmöglichkeiten (0–4 Punkte) und beurteilt die Gelenkfunktion und die Schmerzsituation nach Gelenkersatz. Je höher der kumulierte Wert, desto besser das funktionelle Ergebnis und desto geringer das Schmerzniveau und die Einschränkungen in der Alltagsaktivität.

Der UCLA-Score (University of California, Los Angeles activity scale) besteht aus nur einer Frage mit zehn Antwortmöglichkeiten, von denen eine auszuwählen ist (1–10 Punkte). Die Bandbreite reicht von „Ich bin komplett inaktiv, abhängig von anderen und bettlägerig“ über „Ich betreibe regelmäßig leichte Aktivitäten wie Laufen, begrenzte Tätigkeiten im Haushalt, uneingeschränkte Einkaufsgänge“ bis hin zu „Ich betreibe regelmäßig Sportarten mit Stoßbelastung“. Der UCLA-Score basiert also auf der höchstmöglichen klassifizierten Aktivität, unabhängig von deren Häufigkeit oder Intensität.

Der Knee Society Score (KSS) ist in zwei Teile mit je 100 Punkten unterteilt. Der „Knee Score“ bewertet Schmerz, Beweglichkeit und Stabilität des Kniegelenks während der „Functional Score“ die Fähigkeiten des Gehens und Treppensteigens abbildet [10, 16].

Der WOMAC-Score (Western Ontario and McMaster Universities Osteoarthritis Index) wurde in den 1980er-Jahren in Kanada entwickelt und umfasst 24 Fragen, welche die Dimensionen Schmerz (5 Fragen), Steifheit (2 Fragen) und Alltagsaktivitäten (17 Fragen) abdecken. Im Unterschied zur ursprünglichen Version kommt im Rahmen unserer klinikinternen Nachsorge eine abgewandelte Form zur Anwendung, in der 11 Antwortmöglichkeiten (0–10 Punkte) bestehen, sodass bis zu 240 Punkte erreicht werden können. Zudem wurde jeweils der Kehrwert berechnet und angegeben, sodass bei hohen Ergebnissen entgegen dem Original eine gute Gelenkfunktion vorliegt.

Neben der dargestellten schriftlichen Befragung wurden alle Patienten zu einer standardisierten Nachuntersuchung eingeladen. Diese wurde frühestens 6 Monate postoperativ durchgeführt, damit eventuell persistierende operationsassoziierte Beschwerden die Ergebnisse nicht verfälschten. Die Untersuchung umfasste die Erfassung des Bewegungsumfangs, der Beugefähigkeit und eines möglichen Streckdefizits.

Die statistische Auswertung erfolgte mit Graph Pad Prism 6 (GraphPad Software, Boston, MA, USA) und Microsoft Excel 2010 (Microsoft Corporation, Redmond, WA, USA). Zur vergleichenden Berechnung der Ergebnisse der drei Gruppen verwendeten wir die nicht parametrische One-way ANOVA (Kruskal-Wallis-Test). Zum Vergleich nominalskalierter Daten (z. B. Geschlechterverteilung) kam der Chi-Quadrat-Test zur Anwendung. Das Signifikanzniveau beträgt 5 % (*p* < 0,05).

## Ergebnisse

Weder in Matching 1 noch in Matching 2 unterschieden sich die einzelnen Gruppen bezüglich Follow-up-Periode (*p* = 0,702) oder Alter beim Follow-up (*p* = 0,757 bzw. *p* = 0,279) signifikant (Tab. 1).

Etwa 3 Jahre nach der jeweils letzten Operation (Matching 1) war das funktionelle Ergebnis nach Revision des Monoschlittens auf einen Doppelschlitten in jedem Score signifikant schlechter als das nach Primärimplantation des Monoschlittens. 6 Jahre nach Primärimplantation (Matching 2) galt dies noch für den Oxford Knee Score und den WOMAC-Score, nicht jedoch für den ULCA-Score und Knee Society Score (Tab. 2).

**Table 2:** 

	U2T	Matching 1			Matching 2		
		UKA	TKA	P‑Wert	UKA	TKA	P‑Wert
**Oxford**	30,3 SD 12,0 (7,0–45,0)	40,8 SD 5,7 (24,0–46,0)	36,3 SD 10,9 (14,0-48,0)	*0,001* *UKA-TKA: ns* *TKA-U2T: ns* *UKA-U2T: s*	38,5 SD 7,0 (24–47)	36,0 SD 11,8 (7–48)	*0,033* *UKA-TKA: ns* *TKA-U2T: ns* *UKA-U2T: s*
**UCLA**	5,1 SD 1,3 (2,0–7,0)	6,3 SD 1,1 (3,0–9,0)	5,8 SD 1,7 (3,0–10,0)	*0,009* *UKA-TKA: ns* *TKA-U2T: ns* *UKA-U2T: s*	6,0 SD 1,4 (3–9)	5,2 SD 1,9 (3–10)	*0,075* *UKA-TKA: ns* *TKA-U2T: ns* *UKA-U2T: ns*
**KSS**	142,0 SD 37,0 (49,0–187,0)	178,7 SD 24,2 (107,0–200,0)	157,8 SD 39,5 (60,0–200)	*0,001* *UKA-TKA: s* *TKA-U2T: ns* *UKA-U2T: s*	169,9 SD 26,3 (107–200)	156,9 SD 49,6 (40–200)	*0,687* *UKA-TKA: ns* *TKA-U2T: ns* *UKA-U2T: ns*
**WOMAC**	65,4 SD 27,7 (10,0–96,7)	91,3 SD 12,6 (50,4–100,0)	78,3 SD 24,8 (13,7–100,0)	*<* *0,001* *UKA-TKA: ns* *TKA-U2T: ns* *UKA-U2T: s*	84,9 SD 16,0 (50,4–100,0)	79,5 SD 24,5 (13,3–100,0)	*0,016* *UKA-TKA: ns* *TKA-U2T: ns* *UKA-U2T: s*

*KSS* Knee Society Score, *SD* Standardabweichung, *TKA* bikondylärer Kniegelenksersatz, *U2T* Konversion von einem unikondylären Kniegelenksersatz auf einen bikondylären Kniegelenksersatz, *UCLA* University of California, Los Angeles activity scale,* UKA* unikondylärer Kniegelenksersatz, *WOMAC* Western Ontario and McMaster Universities Osteoarthritis Index

Demgegenüber war das Ergebnis eines auf einen Doppelschlitten revidierten Monoschlittens in keinem der untersuchten Scores dem primären Doppelschlitten signifikant unterlegen, weder im Matching 1 noch im Matching 2. Allerdings lagen die Durchschnittswerte nach primärer Implantation eines Doppelschlittens stets über denen der revidierten Monoschlitten (Tab. 2).

Die Durchschnittswerte des primären Doppelschlitten lagen dabei stets zwischen denen der beiden anderen Gruppen, was jedoch lediglich im Matching 1 und nur im Knee Society Score zu einer signifikanten Überlegenheit der Mono- gegenüber der Doppelschlitten führte. Zum zweiten Follow-up, d. h. ca. 6 Jahre nach Primärimplantation, ließ sich kein signifikanter Unterschied zwischen primärer Mono- und Doppelschlitten mehr festhalten.

**Figure 1:**
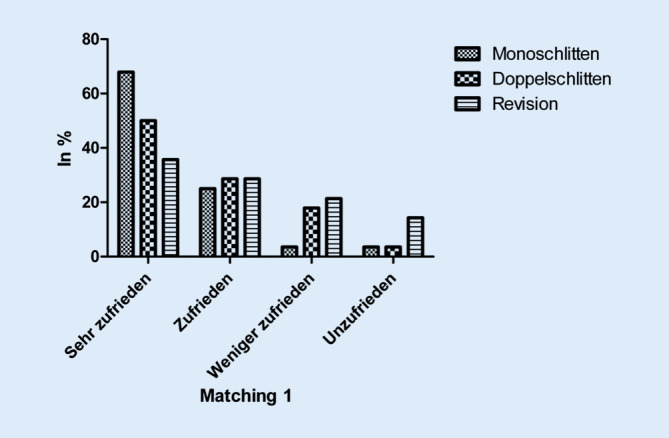

Etwa 3 Jahre nach der jeweils letzten Operation waren fast doppelt so viele Monoschlitten-Patienten mit dem postoperativen Ergebnis sehr zufrieden wie in der Revisions-Gruppe (67,9 % vs. 35,7 %). In der Doppelschlitten-Gruppe war jeder zweite Patient mit dem postoperativen Ergebnis sehr zufrieden. Weniger oder unzufrieden waren 7,2 % der Monoschlitten-Patienten, 21,4 % der Doppelschlitten-Patienten und 35,7 % der Revisions-Patienten (*p* = 0,859). Damit war der Anteil der unzufriedenen Patienten in der Revisions-Gruppe tendenziell höher als in den beiden anderen Gruppen (Abb. 1). Der postoperative allgemeine Gesundheitsstatus wurde von 92,9 % der Monoschlitten-Patienten, 82,1 % der Doppelschlitten-Patienten und 57,1 % der Revisions-Patienten postoperativ als verbessert bewertet (*p* = 0,723).

**Figure 2:**
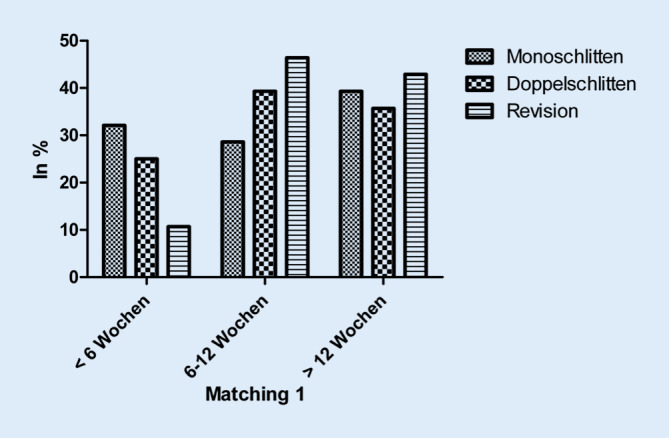

**Figure 3:**
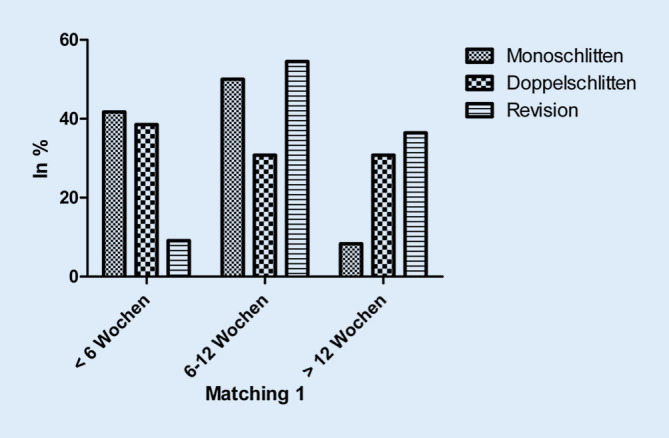

Während 32,1 % der Monoschlitten- und 25,0 % der Doppelschlitten-Patienten ihre Alltagsaktivitäten innerhalb von 6 Wochen wieder vollständig aufnahmen, gelang dies nur 10,7 % der Revisions-Patienten. Letztere benötigten in 46,6 % der Fälle 6–12 Wochen und in 42,9 % der Fälle sogar länger als 12 Wochen (*p* = 0,739; Abb. 2). Die Rückkehr in den Beruf gelang den Patienten der Monoschlitten-Gruppe ebenfalls tendenziell vor jenen der Doppelschlitten- und Revisions-Gruppe (Abb. 3).

**Figure 4:**
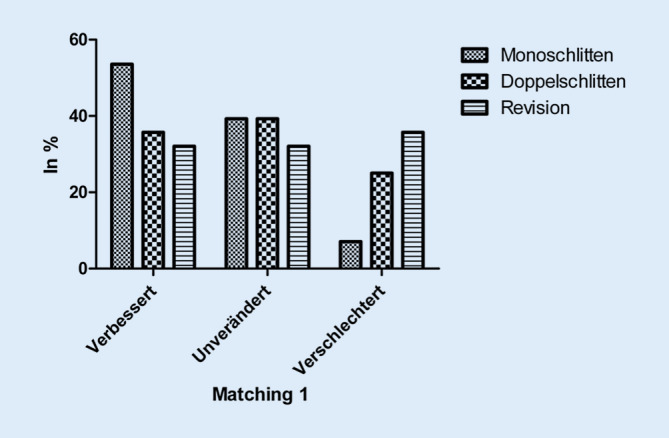

Die Sportfähigkeit wurde von 53,6 % der Monoschlitten-, 35,7 % der Doppelschlitten- und 32,1 % der Revisions-Patienten postoperativ besser eingeschätzt als präoperativ. Demgegenüber gaben 35,7 % der Revisions-Patienten, 25,0 % der Doppelschlitten-Patienten und lediglich 7,1 % der Monoschlitten-Patienten eine Verschlechterung ihrer Sportfähigkeit an (*p* = 0,752; Abb. 4). Der Anteil sog. Low-Impact-Sportarten stieg postoperativ in der Monoschlitten-Gruppe von 88,0 % auf 91,7 % (*p* = 0,672), in der Doppelschlitten-Gruppe von 85,2 % auf 94,4 % (*p* = 0,333) und in der Revisions-Gruppe von 94,4 % auf 100,0 % (*p* = 0,388). Der Anteil der Patienten, die mehrmals die Woche Sport trieben, stieg in der Monoschlitten-Gruppe von 61,5 % auf 84,6 % (*p* = 0,185), in der Doppelschlitten-Gruppe von 47,6 % auf 60,0 % (*p* = 0,463) und in der Revisions-Gruppe von 50,0 % auf 71,4 % (*p* = 0,232). Damit erreichte dieser tendenzielle Anstieg in keiner Gruppe das Signifikanzniveau.

Der Anteil der Patienten mit Streckdefizit im Matching 1 unterschied sich nicht signifikant zwischen den Gruppen (*p* = 0,777). Er betrug bei dem einen Patienten mit Monoschlitten jedoch > 10°, während er bei den drei Patienten mit Doppelschlitten und den zwei revidierten Patienten stets unter 10° lag. Die durchschnittliche Flexionsfähigkeit lag mit 124° in der Monoschlitten-Gruppe und 116° in der Doppelschlitten-Gruppe signifikant über der der Revisions-Gruppe mit 105° (*P* < 0,0001). Der Unterschied zwischen Mono- und Doppelschlitten war hierbei nicht signifikant. Der Anteil der Patienten mit einer Flexionsfähigkeit von weniger als 100° betrug in der Monoschlitten-Gruppe 0,0 % in der Doppelschlitten-Gruppe 11,7 % und in der Revisions-Gruppe 28,0 % der Patienten.

Die Ergebnisse auf Basis des Matching 2, d. h. ca. 6 Jahre nach Primärimplantation, zeigte ein ähnliches Bild bezüglich des funktionellen Ergebnisses: Hier waren 60,7 % der Monoschlitten-Patienten mit dem postoperativen Zustand sehr zufrieden, 46,4 % der Doppelschlitten-Patienten und 35,7 % der revidierten Patienten. Weniger zufrieden oder unzufrieden waren 7,2 % der Monoschlitten-Patienten, 3,6 % der Doppelschlitten-Patienten und 35,7 % der Revisions-Patienten (*p* = 0,981). Eine Verbesserung des allgemeinen Gesundheitszustands wurde von 85,7 % der Monoschlitten-, 96,4 % der Doppelschlitten- und 57,2 % der Revisions-Patienten angegeben (*p* = 0,705).

Im Rahmen des Matching 2 wurde die Sportfähigkeit von 46,4 % der Monoschlitten-, 38,5 % der Doppelschlitten- und 32,1 % der Revisions-Patienten als verbessert bewertet (*p* = 0,859). Der Anteil der sog. Low-Impact-Sportarten stieg bei den Monoschlitten signifikant von 76,2 % auf 100,0 % (*p* = 0,027), bei den Doppelschlitten von 73,9 % auf 93,8 % (*p* = 0,112) und in der Revisions-Gruppe von 94,4 % auf 100,0 % (*p* = 0,388). Der Anteil der mehrmals pro Woche sportlich aktiven Patienten erhöhte sich von 60,0 % auf 66,7 % (*p* = 0,722) in der Monoschlitten-, von 50,0 % auf 64,7 % (*p* = 0,393) in der Doppelschlitten- und von 50,0 % auf 71,4 % in der Revisions-Gruppe (*p* = 0,232). Die Rückkehr in den Beruf gelang den Patienten nach primärer Implantation eines Monoschlittens auch im Matching 2 tendenziell vor jenen nach primärer oder sekundärer Implantation eines Doppelschlittens.

Der Anteil der Patienten mit Streckdefizit unterschied sich auch im Matching 2 nicht signifikant zwischen den Gruppen (*p* = 0,740). Er betrug bei einem Patienten in der Doppelschlitten-Gruppe jedoch > 10°, während er bei dem einzigen Patienten der Monoschlitten-Gruppe und den zwei Patienten der Revisions-Gruppe unter 10° lag. Die durchschnittliche Flexionsfähigkeit lag mit 127° in der Monoschlitten-Gruppe signifikant über der der Doppelschlitten-Gruppe mit 110° und derjenigen der Revisions-Gruppe mit 105° (*P* < 0,0001). Der Unterschied zwischen den beiden bikondylären Gruppen, also Primärimplantat und Revisionsimplantat, erreichte das Signifikanzniveau nicht. Der Anteil der Patienten mit einer Flexionsfähigkeit von weniger als 100° betrug in der Monoschlitten-Gruppe 0,0 % in der Doppelschlitten-Gruppe 14,3 % und in der Revisions-Gruppe 28,0 % der untersuchten Patienten.

## Diskussion

Sowohl der klassische Doppelschlitten als auch der Monoschlitten gelten, bei geeigneter Patientenselektion, als etablierte Therapieoptionen der Pan- bzw. anteromedialen Gonarthrose, die Patienten in ihren körperlichen Aktivitäten erheblich einschränken kann [18, 23]. In der klinischen Praxis ergeben sich insbesondere bei jüngeren, aktiven Patienten regelmäßig Situationen, in denen zwischen den beiden Prothesenkonzepten und ihren Vor- und Nachteilen abgewogen werden muss. In diesem Fall muss den funktionellen Vorteilen des Monoschlittens gegenüber denen eines primären Doppelschlittens auch ihr Nachteil der früheren Lockerung und damit der Fall einer sekundären Konversion auf einen Doppelschlitten gegenübergestellt werden [12, 15, 19].

Diese Studie soll im Rahmen einer Matched-Pair-Vergleichsanalyse die postoperative Funktion sowie die Wiederaufnahme von Alltags-, beruflichen, sportlichen Aktivitäten nach primärem Monoschlitten, Doppelschlitten und Revision eines Mono- auf einen Doppelschlitten beleuchten, um damit eine Entscheidungshilfe im Rahmen des klinischen Abwägens zwischen Mono- und Doppelschlitten bei Grenzindikationen zu liefern. Insbesondere die Wiederaufnahme körperlicher Aktivität nach Wechsel von Monoschlitten hat in der Literatur bislang nur unzureichend Beachtung gefunden.

Das Matching der jeweils 28 Patienten erfolgte zum einen auf Grundlage des Zeitraums zwischen Revisionsoperation und Follow-up-Untersuchung der Revisions-Gruppe (3,2 Jahre, Matching 1). Zum anderen wurde in einem zweiten Matching der Zeitraum zwischen Primärimplantation und Follow-up der Revisions-Gruppe (6,4 Jahre) zugrunde gelegt (Matching 2). Somit repräsentiert das Matching 1 den Zeitraum seit der letzten Operation und zielt damit darauf ab, den Zeitraum zwischen Implantation des aktuellen Gelenks und der Befragung, also den Zeitraum der Gewöhnung an das einliegende Implantat, bei allen drei Gruppen identisch zu wählen. Das Matching 2 zielt darauf ab, die funktionelle Situation eines Patienten in einem konstanten Zeitraum nach der initialen Wahl des jeweiligen Prothesendesigns im Rahmen der Primärimplantation zu eruieren.

Die postoperative Zufriedenheit lag bei den Monoschlitten-Patienten tendenziell höher als in der Doppelschlitten-Gruppe, gefolgt von der Revisions-Gruppe. Auch eine Verbesserung des allgemeinen Gesundheitszustands wurde nach Primärimplantationen tendenziell häufiger beschrieben als nach Wechseloperationen. Diese Unterschiede erreichten in Anbetracht der geringen Fallzahl jedoch nicht das Signifikanzniveau.

Entsprechend schnitten die Monoschlitten-Patienten in allen vier untersuchten Scores tendenziell am besten ab, gefolgt von den Doppelschlitten- und Revisions-Patienten. Im Vergleich der beiden primären Prothesenmodelle zeigten sich bei der betrachteten Fallzahl mit einer Ausnahme jedoch keine signifikanten Unterschiede, sodass die in der Literatur häufig beschriebene funktionelle Überlegenheit der Schlittenprothese auf Grundlage dieser Untersuchung letztlich nicht untermauert werden kann [1, 6, 15, 18, 23]. Ebenfalls fand sich in keinem Score der Nachweis einer signifikanten Unterlegenheit der Revisionsprothese gegenüber einem primär implantierten bikondylären Oberflächenersatz, und zwar weder 3,2 Jahre nach der letzten Operation (Matching 1), noch 6,4 Jahre nach initialer Implantation (Matching 2). Demgegenüber berichten zwei Metaanalysen aus dem Jahr 2018 von Zuo et al. und Sun und Su von schlechteren klinischen Ergebnissen nach Wechsel eines Monoschlittens auf einen bikondylären Oberflächenersatz als nach primärer Implantation eines bikondylären Oberflächenersatzes [27, 30]. Auch Lundebourg et al. (2015) sehen Nachteile in Bezug auf Lebensqualität und Funktionsfähigkeit nach Revision eines Monoschlittens im Vergleich zur Primärimplantation eines bikondylären Oberflächenersatzes [17].

Im Matching 1, also 3,2 Jahre nach der jeweils zuletzt durchgeführten Operation zeigte die Revisions-Gruppe in jedem der vier angewandten Funktions-Scores jedoch signifikant schlechtere Ergebnisse als die Monoschlitten-Gruppe. Anzumerken ist hierbei, dass alle Revisionspatienten mit Standardimplantaten entsprechend eines primären Doppelschlittens versorgt wurden. 6,4 Jahre nach Primärimplantation (Matching 2) zeigten sich signifikante Unterschiede zwischen Monoschlitten- und Revisions-Gruppe noch in zwei der vier Scores. Für die klinische Praxis ist daraus abzuleiten, dass die Indikation zum Wechsel eines Monoschlittens trotz vermeintlicher Einfachheit der Operation keinesfalls leichtfertig zu stellen ist [14, 25].

Die Rückkehr in den beruflichen und privaten Alltag verlief nach Monoschlitten-Implantation tendenziell am schnellsten, gefolgt vom primären Doppelschlitten. Insgesamt benötigte die Revisions-Gruppe sowohl im beruflichen als auch im privaten Bereich jeweils die längste Zeit. Auch in einer Untersuchung von Kievit et al. kehren Monoschlitten-Patienten postoperativ deutlich früher an ihren Arbeitsplatz zurück. Signifikante Unterschiede mit Blick auf Arbeitsfähigkeits-Scores oder postoperative Zufriedenheit fanden sich hingegen nicht [11].

Die Verbesserung der postoperativen Sportfähigkeit war bei den Monoschlitten-Patienten tendenziell höher ausgeprägt als bei den Doppelschlitten- und den Revisions-Patienten. Diese Ergebnisse stehen in Einklang mit einer Metaanalyse von Witjes et al. [28] und eigenen früheren Ergebnissen [21, 22].

Die allgemeine postoperative Sportfähigkeit wurde von der größten Zahl der Monoschlitten-Patienten als besser, der Doppelschlitten-Patienten als unverändert und der Revisions-Patienten als schlechter beurteilt. Besonders auf den letzten Aspekt sollte aus Sicht der Autoren mit dem Patienten vor Durchführung eines operativen Eingriffs im Sinne einer realistischen Erwartungsbildung hingewiesen werden. Der Anteil jener Patienten, die mehrmals pro Woche sportlich aktiv sind, stieg tendenziell in allen untersuchten Gruppen an. Ebenfalls zeigte sich eine Tendenz über alle untersuchten Gruppe zugunsten sog. Low-Impact-Sportarten wie Fahrradfahren, Schwimmen oder Nordic Walking unter Abnahme von sog. High-Impact-Sportarten wie Tennis, Fußball oder Skifahren. Grundsätzlich sind sowohl mit Mono- als auch mit Doppelschlitten sowohl Low- als auch High-Impact-Sportarten möglich [28].

Unsere Ergebnisse zeigen sich vereinbar mit denen von Ho et al., deren Untersuchungen keinen signifikanten Unterschied zwischen Mono- und Doppelschlitten bezüglich der Anzahl der Patienten zeigte, die ihre sportliche Aktivität wiederaufnahmen. Allerdings erfolgte die Wiederaufnahme sportlicher Aktivitäten bei Monoschlitten-Patienten schneller als bei Doppelschlitten-Patienten und erstere zeigten bessere postoperative Funktions-Scores [8].

In einer Studie von Hopper und Leach zeigten Patienten nach Implantation eines Monoschlittens eine signifikant höhere Wiederaufnahmerate sportlicher Aktivität als nach Versorgung mittels klassischem Doppelschlitten. Zudem nahmen Monoschlitten-Patienten ihre sportliche Aktivität früher wieder auf und gingen ihrem Sport häufiger und länger nach [9]. Auch nach Harbourne et al. kehren Patienten, die mit einem Monoschlitten versorgt wurden, mit größerer Wahrscheinlichkeit zu der gewünschten Aktivität zurück als diejenigen, die mit einem Doppelschlitten versorgt wurden [7].

Der vermehrte Bewegungsumfang des Mono- gegenüber dem Doppelschlitten sowie die tendenziell verstärkte und frühere Aufnahme körperlicher Aktivitäten ist vereinbar mit den Ergebnissen von Kleeblad et al. [12].

Gemäß einer Studie von Canetti et al. verkürzt eine roboterassistierte Implantation der deutlich selteneren lateralen Schlittenprothese die Zeit bis zur Rückkehr zum Sport im Vergleich zur konventionellen Operationstechnik signifikant [4]. Eine genauere Positionierung des Implantats durch ein robotergestütztes System konnte für mediale und laterale Monoschlitten dargelegt werden [2].

## Schlussfolgerungen

Die funktionellen Ergebnisse eines konvertierten Monoschlittens zeigen sich denen der Primärimplantation eines Monoschlittens auf Basis des 3‑Jahres-Follow-ups signifikant unterlegen. Zudem ist der Bewegungsumfang der Doppelschlitten geringer als der des Monoschlittens. Die Rückkehr in Sport, Beruf und Alltagsaktivitäten dauerte nach Revision tendenziell länger als nach Primärimplantation eines Mono- oder Doppelschlittens, was die Lebensqualität und die Möglichkeit, aktiv an der Gesellschaft teilzunehmen, reduziert. Diese Informationen können helfen, die Vor- und Nachteile abzuwägen und die beste Behandlung und den besten Zeitpunkt für die Patienten zu wählen.

## Abkürzungen

BMI: Body-Mass-Index
KKS: Knee Society Score
PROM: Patient Reported Outcome Measures
SD: Standardabweichung
TKA: Total Knee Arthroplasty/Bikondylärer Kniegelenkersatz
U2T: Konversion von einem unikondylären Kniegelenkersatz auf einen bikondylären Kniegelenkersatz
UCLA: University of California, Los Angeles
UKA: Unicompartmental Knee Arthroplasty/Unikondylärer Kniegelenkersatz
WOMAC: Western Ontario and McMaster Universities Osteoarthritis Index
